# On the need to better specify the concept of “control” in brain-computer-interfaces/neurofeedback research

**DOI:** 10.3389/fnsys.2014.00171

**Published:** 2014-09-29

**Authors:** Guilherme Wood, Silvia Erika Kober, Matthias Witte, Christa Neuper

**Affiliations:** ^1^Department of Psychology, Karl-Franzens-University GrazGraz, Austria; ^2^BioTechMedGraz, Austria

**Keywords:** BCI, neurofeedback, executive functions, dual-process theory, rumination, meta-cognition, cognitive strategies

## Abstract

Aiming at a better specification of the concept of “control” in brain-computer-interfaces (BCIs) and neurofeedback (NF) research, we propose to distinguish “self-control of brain activity” from the broader concept of “BCI control”, since the first describes a neurocognitive phenomenon and is only one of the many components of “BCI control”. Based on this distinction, we developed a framework based on dual-processes theory that describes the cognitive determinants of self-control of brain activity as the interplay of automatic vs. controlled information processing. Further, we distinguish between cognitive processes that are necessary and sufficient to achieve a given level of self-control of brain activity and those which are not. We discuss that those cognitive processes which are not necessary for the learning process can hamper self-control because they cannot be completely turned-off at any time. This framework aims at a comprehensive description of the cognitive determinants of the acquisition of self-control of brain activity underlying those classes of BCI which require the user to achieve regulation of brain activity as well as NF learning.

## Introduction

Brain computer interfaces (BCIs) make possible the direct communication pathway between the brain and an external device. BCIs are often directed at assisting, augmenting, or repairing human cognitive or sensory-motor functions. Individuals learn how to induce certain patterns of brain activity, which can be detected and transcoded into some form of action or feedback in the external device. One special case of BCI is neurofeedback (NF), in which the aim is not to control an external device but rather to use external feedback to modulate specific aspects of physiological signal intrinsic to the brain. Both human and nonhuman animals are able to learn to use BCI/NF with a short amount of training (Sterman, 1977; Nicolelis and Lebedev, 2009; Phillippens and Vanwersch, 2010).

Generally, the term “BCI control” has been used interchangeably to refer to two different processes. On the one side, “BCI control” refers to the ability to control an external device and can be seen mainly as a complex problem of neuroengineering (Donoghue, 2008). This definition simultaneously involves neuro-bio-psychological, data analytical and ergonomical aspects (see Kübler et al., 2011). On the other side, “BCI control” may refer to the much more specific ability of an individual to control some aspects of his/her own brain activity (Hinterberger et al., 2003; Halder et al., 2011), which is clearly a neurocognitive topic that is central but not restricted to BCI/NF. Broadly speaking, not only BCI/NF but many other processes such as meditation techniques (Tang et al., 2014), emotion regulation (Thayer and Lane, 2000) and even psychotherapy (Beauregard, 2007) also induce some form of self-control of brain activity. Since the definition of “BCI control” from either a neuroengineering perspective or from a neurocognitive perspective fundamentally differs, it is necessary to disentangle both views. The topic of the present article is “BCI control” as self-regulation of neuronal activity and, for the sake of transparency, it will be called hereafter “self-control of brain activity”.

With the aim of better understanding BCI/NF learning, the first step to characterize “self-control of brain activity” is to specify the cognitive mechanisms responsible for learning control. The more popular models of BCI/NF discuss “operant conditioning” and a “motor skill learning” as these mechanisms (Hammer et al., 2012). However, many studies indicate that other cognitive mechanisms such as locus of control towards technology (Burde and Blankertz, 2006; Ninaus et al., 2013; Witte et al., 2013), aptitude towards BCI (Hammer et al., 2012; Halder et al., 2013), motivation (Kleih et al., 2010) and spontaneous strategies (Kober et al., 2013) also influence BCI or NF learning. As a consequence, these predictors may either constitute a secondary correlate of self-control of brain activity or may represent key cognitive processes in addition to conditioning and skill learning. Given the high variety of cognitive and emotional processes apparently associated with self-control of brain activity and BCI learning, it is particularly useful to define a simple but comprehensible framework to evaluate the common and unique contributions of each one of these processes.

A dual-processes theory has been related to BCI/NF learning (Lacroix, 1986; Hammer et al., 2012). In the following, we shortly point out how this theory can be employed to better understand how the processes mentioned above might determine self-control of brain activity.

## Two types of mental activity

The dual-processes theory categorizes the whole mental activity into two main types of processing: more automatic and capacity-free processes (i.e., type I processes) vs. more controlled and capacity-limited processes (i.e., type II processes). Type I processes reflect the automatic, capacity-free, effortless and context-specific information processing such as for instance trying to open the office door with the home key because one has been thinking about dinner. Moreover, type I processes are usually unconscious and difficult to control by self-instruction. Type II processes reflect the activity of a supervisory attention system, specialized in monitoring and regulating the activity in other cognitive systems (Shallice and Cooper, 2011). Type II processes are usually in the center of our focus of attention (but see Horga and Maia, 2012 for an exception), are regulated mainly by self-instruction and are fundamental for executive functions and metacognitive abilities (Bewick et al., 1995). Accordingly, control beliefs are much more related to the function of the type II processes while the heuristics regulating most of our cognitive activity and behavior are type I processes.

A central aspect of the dual-processes theory is that both automatic and controlled processes have control of behavior as well as of different aspects of cognition (Alos Ferrer, 2013) but both learn from and react to different aspects of the task at hand. Automatic systems learn only through cumulative reward while controlled systems are more flexible, context-oriented and learn fast from instructions. It is beyond the scope of this perspective article to review every single manifestation of automatic vs. controlled processing to each one of the predictors of self-control of brain activity. Instead, we present one example regarding motivation, which may suffice to make our point: motivation consists of a more controlled component called intrinsic motivation, which is highly sensitive to self-instruction and self-efficacy beliefs, and a more automatic component called extrinsic motivation, which is more sensitive to the current amount of reward received (Ryan and Deci, 2000). Accordingly, as long as some reward can be obtained during BCI/NF learning, automatic processing will predominate. Controlled processing will be engaged when negative feedback predominates over longer periods of time and will have a larger impact, if the participant shows high levels of intrinsic motivation. In summary, dual-process models such as Lacroix (1986) make clear that self-regulation is not a unitary process but rather the result of the conjoint action of type I and type II processes.

## A framework of self-control of brain activity

Automatic and controlled processes determine self-control of brain activity in very different ways. Even more, not every cognitive process is necessary and sufficient to perform a specific BCI/NF task, but instead a small subset may play a key role. The remaining mental activity -that is neither necessary nor sufficient for a specific BCI/NF task -will act on BCI/NF learning in one of two ways: firstly, this activity can interfere with the learning process, if it hampers self-control of the specific aspect of brain activity being targeted in a specific BCI/NF task. Secondly, activations can promote the learning process indirectly, if they do not interfere with the activity in that small subset of both automatic and controlled processes necessary and sufficient to perform the BCI/NF task at hand. Although in some BCI classes such as those employing electrocorticogram or other kinds of stable and specific brain signals such as SSVEP the influence of unspecific processes signal is barely important, cognitive BCIs (Astrand et al., 2014) or BCI classes based on cognitive tasks such as mental calculation and motor imagery (Halder et al., 2011; Hammer et al., 2012) should be more subjected to the effects of different forms of self-control over brain activity. Based on the differentiation between automatic vs. controlled processing as well as necessary vs. unnecessary processes, we define a framework of self-control of brain activity.

We start with the automatic and controlled processes both necessary and sufficient to perform BCI/NF tasks: both are subsumed under *local control network*. The more the feedback provided by BCI/NF reflects the activity in these networks, the more efficient is the learning process. The role of automatic and controlled processes in the *local control network* is complementary: automatic processes are driven directly by the amount and quality of feedback obtained whereas controlled processes are driven by the verbalizations and self-instruction (Lacroix, 1986), that are largely under conscious control of the individual and subjected to beliefs and expectations. While automatic learning is very insensitive to verbal instructions and only takes place when some pattern of reaction is systematically rewarded, controlled processes are mainly driven by direct verbal instructions. Efficient BCI/NF learning reflects the timely combination of both processes depending on the present learning rates: when a steep learning curve is forming, automatic processes take the lead, when the learning curve temporarily flattens, controlled processes correct the course by means of self-instruction (Lacroix, 1986). The optimal level of self-control of brain activity in the *local control network* is achieved under two main conditions: (i) avoidance of irrelevant associations between internal states and external reward; and (ii) staying engaged and focused on the task at hand without distractions. As we will discuss below, condition (i) can be achieved when activity in the *organismic control network* is reduced to a minimum and condition (ii), when the *central control network* frees the most of its limited resources for the *local control network*.

**Figure 1:**
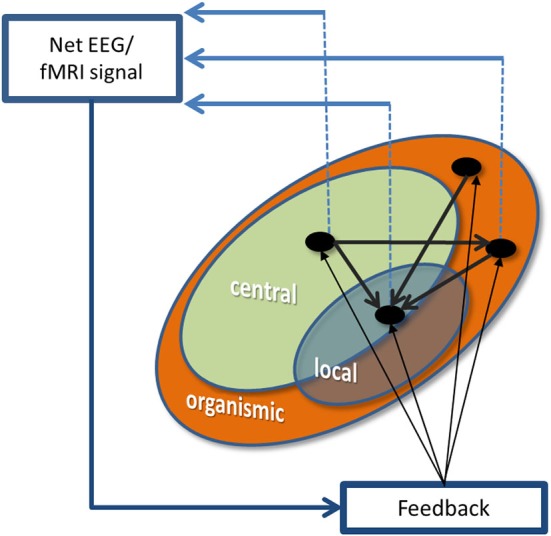
**A schema of different types of self-control of the brain activity**. Specific cognitive processes such as for instance motivation, mood, attention and executive functions are represented by black dots. Black arrows connecting the dots represent the interactions between the different cognitive functions. Two dots are depicted over the domain of organismic control network to illustrate that these processes are largely independent from one another. The contribution of the three types of self-control to physiological signals is represented by the dashed lines linking the specific cognitive processes to the physiological signals being recorded for BCI/NF learning.

We define a framework of three concentric circles (Figure 1) representing three sources of self-control. First, the outmost and thus most unspecific level of response to feedback reflects basically automatic processes. Second, the middle circle depicts central control networks performing controlled processing. Finally, in the innermost level, we describe networks responding specifically to the BCI/NF learning protocol. This local control relies on both automatic and controlled processes.

We define those *automatic* processes unnecessary to perform a given BCI/NF task as the *organismic control network*. We call them organismic control because it reflects the activity of the thousands of automatic and unconscious mental processes regulating the largest part of cognitive activity (Dijksterhuis and Nordgren, 2006). The interference of these processes is high when unnecessary automatic reactions to feedback are triggered, which compete with the learning process taking place in the *local control network*. Rumination, for instance, describes the intrusion of negative feelings about past experiences in the stream of thoughts and emerges primarily during relaxation (Nolen-Hoeksema et al., 2008). The intrusion of ruminative thoughts is an example of the negative impact of the *organismic control network* on self-control of brain activity during BCI/NF learning. Cognitive processes subsumed under *organismic control network* are not easily influenced by direct instructions and mostly not even conscious to the participant. Therefore, it may be very important to monitor any signs of negative influences originating in organismic control networks. This unwanted activity should be fed back in a timely manner during training. As a consequence, processes like increased anxiety or intrusive thoughts are accessible to BCI/NF users and can trigger appropriate learning mechanisms capable to control or suppress these processes.

Finally, we define the *central control network* as those *controlled* processes not strictly necessary to perform a given BCI/NF task. Controlled processes have limited capacity, so that every bit of irrelevant information being employed in the *central control network* will be missed by the *local control network*. The negative impact of *central control network* is high when improper strategies, self-instruction, over-instruction or excessive attention to the self (Leary et al., 2006) withdraw resources from the *local control network* and hamper the regulation of the learning process in a similar way as a dual-task (Logan and Gordon, 2001) drains resources. In contrast to the *organismic control network*, controlled processing is largely under conscious control and can be modulated directly by instructions (Dijksterhuis and Nordgren, 2006).

In summary, the aim of any BCI/NF learning is to magnify the signal produced by *local control networks* and suppress as much as possible the activity elsewhere. To do that, it is in our view necessary to take into consideration the specificities of two types of cognitive activity subsumed under *organismic control networks* and *central control networks*, since they imply very different learning mechanisms sensitive to different types of cues and reward. On the one side, participants should learn to decouple irrelevant from those relevant automatic processes. One way to achieve this is to monitor the automatic processes regulating for instance negative emotional reactions and anxiety as well as with a more selective schedule of reward and punishment. On the other side, participants should learn to use the *central control networks* to suppress irrelevant cognitive activity operating under conscious control such as excessive attention to the self (Leary et al., 2006). This can be achieved by direct instruction or self-instruction. Once this balance is achieved, the outcome of BCI/NF learning should be improved. Among many other possibilities, one simple experiment to investigate how the suppression of irrelevant cognitive activity could improve BCI/NF learning would involve the monitoring of inner speech (Perrone-Bertolotti et al., 2014). Extra feedback requiring focus on the concrete task is presented when an increase in the levels of inner speech is detected in combination with a local flattening of the learning curve. As suggested by Leary et al. (2006), this extra feedback should help to reduce excessive attention to the self and improve learning.

## Final remarks

We propose that self-regulation of brain activity should be distinguished from the more general process of “BCI learning”, since the latter one is more of a neuroengineering problem whereas the former is mainly a neurocognitive problem. To better understand how self-regulation of brain activity works, we propose to look at the cognitive predictors of BCI/NF performance from the point of view of a framework which organizes them according to the main type of cognitive processing required: more automatic or more controlled processing. Further on, we distinguish between cognitive resources necessary and sufficient for BCI/NF learning and other cognitive processes, which should be suppressed or down-regulated to improve learning. Finally, we argue that our framework can be very useful to optimize BCI learning, since it predicts the most suitable tools to modulate the activation generated by automatic and controlled cognitive processes.

## Conflict of interest statement

The authors declare that the research was conducted in the absence of any commercial or financial relationships that could be construed as a potential conflict of interest.
